# Prediction of serine phosphorylation sites mapping on *Schizosaccharomyces Pombe* by fusing three encoding schemes with the random forest classifier

**DOI:** 10.1038/s41598-022-06529-5

**Published:** 2022-02-16

**Authors:** Samme Amena Tasmia, Md. Kaderi Kibria, Khanis Farhana Tuly, Md. Ariful Islam, Mst Shamima Khatun, Md. Mehedi Hasan, Md. Nurul Haque Mollah

**Affiliations:** 1 Bioinformatics Laboratory, Department of Statistics, University of Rajshahi, Rajshahi, 6205 Bangladesh; 2 Department of Microbiology and Immunology, Tulane University School of Medicine, Tulane University, New Orleans, LA 70112 USA; 3 Tulane Center for Biomedical Informatics and Genomics, Division of Biomedical Informatics and Genomics, John W. Deming Department of Medicine, School of Medicine, Tulane University, New Orleans, LA 70112 USA

**Keywords:** Computational biology and bioinformatics, Molecular biology, Mathematics and computing

## Abstract

Serine phosphorylation is one type of protein post-translational modifications (PTMs), which plays an essential role in various cellular processes and disease pathogenesis. Numerous methods are used for the prediction of phosphorylation sites. However, the traditional wet-lab based experimental approaches are time-consuming, laborious, and expensive. In this work, a computational predictor was proposed to predict serine phosphorylation sites mapping on *Schizosaccharomyces pombe *(*SP*) by the fusion of three encoding schemes namely k-spaced amino acid pair composition (CKSAAP), binary and amino acid composition (AAC) with the random forest (RF) classifier. So far, the proposed method is firstly developed to predict serine phosphorylation sites for *SP*. Both the training and independent test performance scores were used to investigate the success of the proposed RF based fusion prediction model compared to others. We also investigated their performances by 5-fold cross-validation (CV). In all cases, it was observed that the recommended predictor achieves the largest scores of true positive rate (TPR), true negative rate (TNR), accuracy (ACC), Mathew coefficient of correlation (MCC), Area under the ROC curve (AUC) and pAUC (partial AUC) at false positive rate (FPR) = 0.20. Thus, the prediction performance as discussed in this paper indicates that the proposed approach may be a beneficial and motivating computational resource for predicting serine phosphorylation sites in the case of Fungi. The online interface of the software for the proposed prediction model is publicly available at http://mollah-bioinformaticslab-stat.ru.ac.bd/PredSPS/.

## Introduction

Various post-translational modifications (PTMs) are associated with almost all biological processes by regulating protein functions. However, the unusual states of PTMs are frequently associated with human diseases. Protein phosphorylation is a reversible post-translational modification (PTM) of proteins in which an amino acid residue is phosphorylated by most commonly serine (S), threonine (T), and tyrosine (Y) in eukaryotes. Approximately there are 13,000 phosphorylated sites in human proteins^1^. Various studies indicate that residues of phospho-serine (S), phospho-threonine (T), and phospho-tyrosine (Y) involve signaling transduction and functional control, as indicated by various studies^2–6^. Around 81% of human diseases are associated with phosphorylation^7^. Both cardiovascular disease and type 2 diabetes (T2D) are significantly associated with serine phosphorylation^8^. In microbial phosphorylation, functional functions and molecular mechanisms have recently been introduced to understand^2,9–13^. A single protein can have many phosphorylation sites, and each cell can have thousands of them. Some results of phosphorylation gone awry can include cancer, and diabetes^10,11^. As the importance of phosphorylation in the perspective of biological protein systems and direction to basic biomedical drug design has increased in recent decades, research on phosphorylation has been developed. *Schizosaccharomyces pombe* (*SP*) is a species of yeast known as fission yeast. It is considered a model organism in molecular and cell biology and is used in traditional brewing. It is a unicellular eukaryote with rod-shaped cell. The *SP* has become a notable model system to study basic principles of a cell that can be used to understand more complex organisms like mammals and in particular humans^14,15^. The PomBase model organism database (MOD) has fully unlocked the power of *SP*, with many genes orthologous to human genes identified as 70% up to now^16,17^, including many genes involved in human disease^17^. The *SP* genes have been linked to fifty human diseases, including cystic fibrosis, genetic deafness, diabetes, and cardiovascular diseases^18^. Cancer-related genes make up the biggest collection of human disease-related genes. Among them, 23 genes are involved in DNA damage and repair, checkpoint controls, and the cell cycle. The *SP*’s utility in studying the activities of genes linked to human disease has been investigated by different research groups^16–18^. The *SP* protein-coding genes that produce products that are comparable to proteins produced by 289 genes that are mutated, amplified, or deleted in human disease have been discovered. There are around 289 human disease-causing mutant genes that produce proteins similar to some proteins of *SP* genes. A total of 172 *SP* proteins have similarity with members of this data set of human disease proteins. The largest groups of human disease-related genes are those implicated in cancer^18^. Therefore, serine phosphorylation site prediction might be played a vital role to understand the molecular mechanisms of some human diseases.

There are several experimental and computational approaches for prediction of protein phosphorylation sites. While researchers do not yet know the phosphorylation specificity mechanism, the initial identification of modified microbial phosphorylation protein sites is therefore paradigmatic in the current era^19,20^. To further illuminate the mechanism of phosphorylation, prediction of microbial phosphorylation sites is essential. Identifying the microbial phosphorylation sites in proteins is a requirement because of the possible importance of microbial phosphorylation and provides useful evidence in biomedical research. The experimental identification of the sites of phosphorylation is important and depends primarily on laborious and costly mass spectrometry analysis. Therefore, computational modeling of microbial phosphorylation sites based on protein sequence information is highly desired before experimental investigation. While a large number of quantitative studies have been performed in higher organisms^21–23^, microbial cell predictions are still uncommon. To date, the prediction of microbial phosphorylation sites^24–26^ has been proposed by two analytical methods. Hasan et al. created the first online ML predictor in 2019^27^ to predict non-specific or general phosphorylation sites in microbes, namely MPSite with a random forest (RF) classifier, which predicts phosphorylated serine (pS) and phosphorylated threonine (pT) residues on the targeted protein sequences. The proteins in each species are well known to have a separate substrate structure for the binding of various protein kinases (PKs). Thus, the prediction precision may be enhanced by developing the ML-based predictors in an organism-specific way. The training dataset consisting of 103 phosphorylated serine (pS) and 37 phosphorylated threonine (pT) sites was prepared by Miller et al. in 2008 and the first bacterial-specific online NetPhosBac 1.0 predictor was created^24^ applying artificial neural network (ANN). Li et al. considered the same pS and pT dataset from NetPhosBac in 2015, and developed a cPhosbac predictor using the support vector machine (SVM) based machine leaning algorithm^25^. The prediction model cPhosBac showed better performance compare to the NetPhosBac predictor. However, so far, there is no any computational method for prediction of serine phosphorylation site mapping on *SP* in the literature. More recently, Tasmia et al. developed an improved lysine succinylation site prediction model for homo sapiens by the fusion of three encoding schemes namely k-spaced amino acid pair composition (CKSAAP), binary and amino acid composition (AAC) with the random forest (RF) based machine leaning approach^28^. Therefore, in this study, an attempt was made to develop a computational predictor to predict serine phosphorylation sites mapping on *SP* by the fusion of those three encoding schemes (binary, CKSAAP, AAC) with the random forest (RF) classifier. In section “Materials and methods”, we have applied the necessary materials and methods for the creation of the proposed computational technique. The summary results and their discussions are given in sections “Results” and “Discussion”, respectively, and the conclusion of this study is provided in “Discussion”.

## Materials and methods

### Data sources and descriptions

The dataset for serine phosphorylated protein sequences mapping on *SP*, was downloaded from the database of Phospho-Sites in Animal and Fungi (dbPAF), which is an updated resource for annotating protein phosphorylation sites in prokaryotes (http://dbpaf.biocuckoo.org/download.php). The dataset was consisted of 860 serine phosphorylated protein sequences with 5633 positive sites and 42,765 negative sites. The phosphorylated positions were referred to as positive sites as seen in other studies^27,29,30^, the resting serine residues are considered as non-phosphorylated sites (negative sites) in the protein chains.

### Data preparation and overview on the development of the proposed prediction model

To prepare the dataset to develop an effective protein PTM site prediction model, it is required to adjust some tuning parameters including CD-HIT cutoff (CHC), protein sequence window size (WS), and positive and negative window ratio^29,31–35^. Different authors used different CHC, WS, and ratios of positive and negative windows to build their prediction models. For example, CHC = 30%, WS = 21, and ratio 1:2 were used by Hasan et al.^27^, CHC = 80%, WS = 21 and ratio 1:2 were used by Chen et al.^36^, CHC = 30%, WS = 56 and ratio 1:2 were used by Hasan et al.^37^, CHC = 40%, WS = 27 and ratio 1:1 were used by Mosharaf et al.^34^ to improve their predictors. In this study, we consider CHC at 30% to remove the redundant sequence from the dataset, since over prediction problem arises due to the redundant sequence^27,38^. After removing the redundant sequences, the reduced dataset consisted of 766 serine phosphorylated protein sequences with 4530 positive sites. Then we created the training dataset by randomly taking 690 (90%) phosphorylated protein sequences with 3925 positive and 33,360 negative window sites. The rest 76 (10%) phosphorylated protein sequences with 605 positive and 3345 negative window sites were considered to create the independent test set. Then we selected WS = 25 by two sample logo (TSL) analysis to generate the effective feature variables for both training and independent test datasets. Each window was identified as a 2*w* + 1 = 25 (*w* = residue peptide segment) length peptide segment with serine (S) in the middle. That is, each window was represented by a 25 (± 12)-residue peptide segment with S in the middle. The total number of positive windows (*n*_*1*_ = 3925) and negative windows (*n*_*2*_ = 33,360) were clearly unbalanced in the training dataset. It has been demonstrated that the statistical learning techniques become computationally intractable and accuracy suffers significantly due to the imbalanced number of individuals between positive and negative groups. Many PTM site prediction studies, including the phosphorylation sites prediction, employ a relatively balanced ratio of observations between the positive and negative groups during the training of the classifiers (e.g., the ratio of positives versus negatives is controlled at 1: 1 or 1: 2)^39–41^ to address this issue. In this study, the training datasets were built at three ratios of 1:1, 1:2 and 1:3 of positive and negative window samples to create a comparatively balanced dataset by randomly taking the negative window samples out of *n*_*2*_ = 33,360 for each ratio case. The 1:1 ratio based training dataset was created by taking all 3925 positive windows and randomly 3925 negative windows out of *n*_*2*_ = 33,360. The 1:2 ratio based training dataset was constructed by taking all 3925 positive samples and randomly 3925 × 2 = 7850 negative samples out of *n*_*2*_ = 33,360. Similarly, the 1:3 ratio based training dataset was constructed by taking all 3925 positive samples and randomly 3925 × 3 = 11,775 negative samples out of *n*_*2*_ = 33,360. For each training dataset, we developed the prediction model and examined their performance by using 5-fold cross-validation (CV) and the independent test.

**Figure 1:**
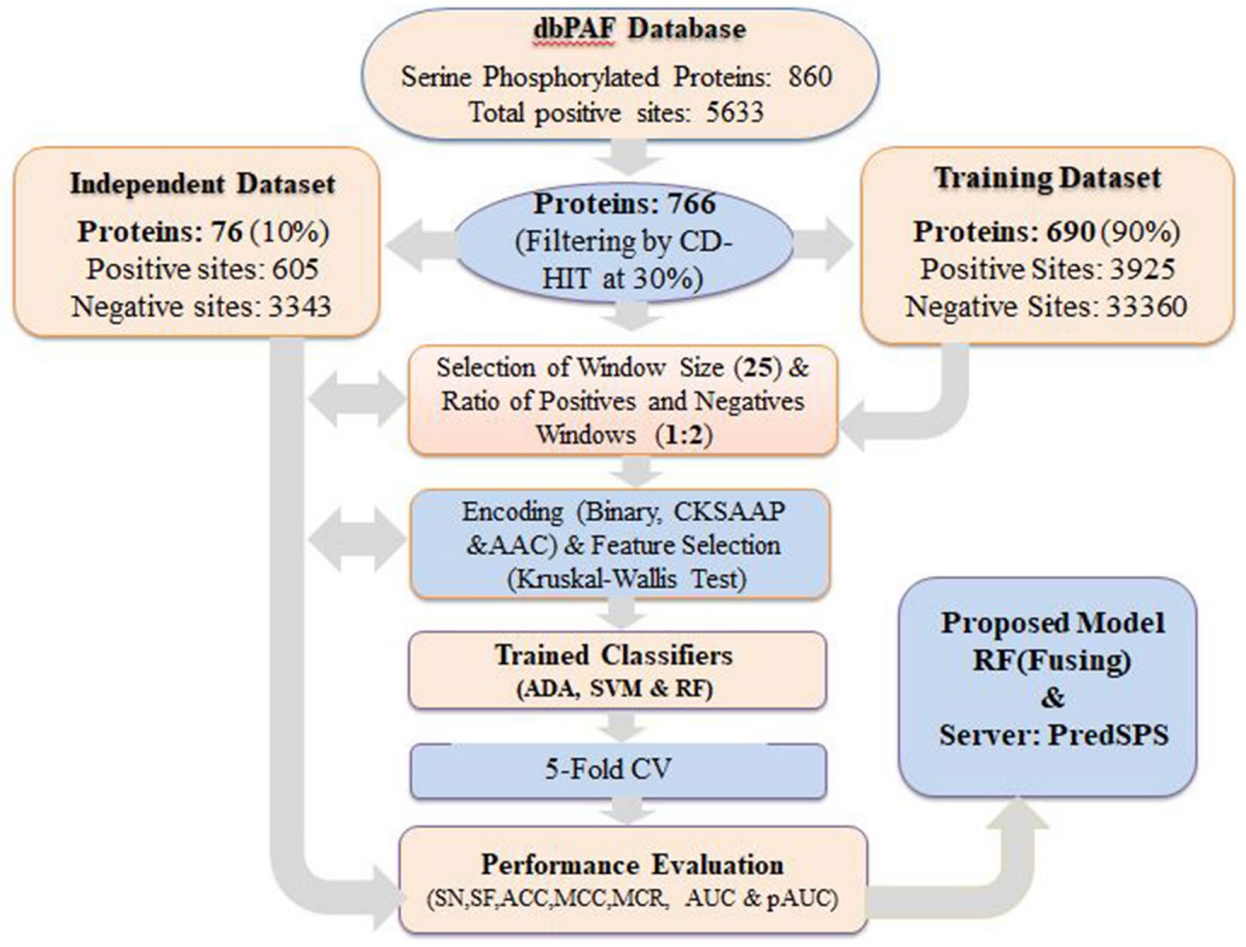
An overview of the proposed PredSPS predictor.

We considered three popular encoding schemes (Binary, CKSAAP, and AAC) to translate the protein window sequence features to numeric features (see section “Data encoding scheme”). Then we used Kruskal–Wallis (KW)^30^ test statistic to select the effective encoder features to develop the prediction models. To pick a better prediction model, we trained three popular classifiers, ADA^42^, SVM^43^, and RF^44^ (see section “Learning classifier”) based on the encoded features of each three schemes, separately. Then we developed an improved prediction model by fusing three encoding schemes with each of ADA, SVM, and RF machine learning approaches (see section “Fusion model”). We observed that the RF based combined model outperform the other alternative candidates. Thus, as seen in Fig. 1, we developed an improved computational prediction model.

### Two sample logo (TSL) analyses

The Two sample logo (TSL) analysis for the protein sequence is used to illustrate the significant differences between the positive and negative window groups of amino acid samples. It finds the difference between the two window groups by identifying the statistically significant residues around the protein PTM site. The residue sample follows the same distribution in both positive and negative window groups for each amino acid at a specific position. Let *X* and *Y* denote two groups of protein sequences based on negative and positive windows. Let |*X*| and |*Y*| denote the number of sequences, and N denotes the length of each window in both groups. Let *X*_*i*_ be the ith sequence in group *A* and let *X*_*i,j*_ is the *j*th position in *A*_*i*_. $t{A}_{X}^{j,r}=1,if{X}_{i,j}=r,{\mathrm{ otherwise }A}_{X}^{j,r}=0,$ where *r* is the symbol of a residue. The vector ${A}_{Y}^{j,p}$ is formed conversely. Then we calculate the *p*-value of H_0_ so that both vectors ${A}_{X}^{j,p}$ and ${A}_{Y}^{j,p}$ follows the same distribution. Two sample t-tests and binomial tests are usually used for testing the null hypothesis H_0_. It should be pointed out that the binomial test is more accurate than the t-test, but the t-test is significantly faster than the binomial test. Two types of graphical image are demonstrated in the TSL analysis: (i) Significant symbols of amino acid are plotted in a display that reflects the size of the symbol that is proportional to the difference of two amino acid samples, (ii) Significant symbols of amino acid are plotted based on the identical size for every amino acid symbols. Amino acids are classified into two groups: (i) enriched samples in the optimistic window, (ii) depleted samples in the optimistic window.

### Data encoding scheme

The protein sequence features are required to convert the numeric features to develop a prediction model. Numerous encoding approaches were developed for converting sequence data to numeric data. In this study, we utilized three popular encoding approaches as described below:

#### CKSAAP encoding

The composition of *k*-spaced amino acid pairs (CKSAAP) is a popular encoding approach for various PTM site predictions^37^. CKSAAP encoding approach has been mostly developed for solving various bioinformatics problems^31–33,37,45–50^.A sequence fragment of 25 amino acids is identified from the phosphorylation or non-phosphorylation sites in the current study. For every single *k* (Gap between two amino acids denoted by *k*), it may construct (21 $\times$ 21) = 441 (21 denotes 21 kinds of amino acids with the gap (O)) kinds of amino acid pairs (i.e., AA, AC, AD,…, OO), if window size of the fragment is $2r+1$. There is 21 × (*k* max + 1) × 21 = 2646 specific combinations of amino acids are produced for a maximum score of k (taking k max = 5). Then the following equation is used to measure the feature vectors:

1 $$\left(\frac{{N}_{AA}}{{N}_{tal}},\frac{{N}_{AC}}{{N}_{tal}},\frac{{N}_{AD}}{{N}_{tal}},\dots ,\frac{{N}_{00}}{{N}_{tal}}\right)441)$$

where ${N}_{tal}$ is the total composition residue length, ${N}_{AA},{N}_{AC},\dots ,{N}_{00}$ denotes the fragment’s frequency of the amino acid pair. More details are available somewhere^51,52^.

#### Binary encoding

The binary encoding approach was used to transform 21 amino acids (including gap (O)) into numeric vectors. Therefore, 21 different amino acids (like ACDEFGHIKLMNPQRSTVWYO) are arranged throughout this encoding scheme. Almost every amino acid is shown by a 21-dimensional binary vector in the query set of proteins. A: 100000000000000000000, C: 010000000000000000000, …, O: 000000000000000000001, etc. The central location is considered as *K* for every window of phosphorylation site to be included in the report. If a window of size is 25, then the entire dimension of the encoding scheme is (21X (25–1)) = 504. Details are described in previous studies^31,32^.

#### AAC encoding

One of the most common and widely used strategies in protein bioinformatics analysis is Amino acid composition (AAC) encoding^53,54^. It can encode amino acid event frequencies to produce protein arrangements data. The amino acid event frequencies in the arrangement regions enclosing the phosphorylation and non-phosphorylation sites (the site itself isn't recorded) were used to calculate AAC in this study. For each group, 20 frequencies for 20 different amino acids were calculated. Given a split arrangement x with a 25-mer string length, *n*_*x*_(*m*) is the quantity of certain amino acid, m, occurring in the section, where m specifies the 20 amino acids. As a result, the probability *P*_*x*_(*m*) of a specific amino acid m is,

2 $$P_{x} \left( m \right) = \frac{{n_{x} \left( {\text{m}} \right){ }}}{{\mathop \sum \nolimits_{m = 1}^{20} n_{x} \left( m \right)}};{\text{ k}} = {1}, \ldots ,{ 2}0$$

Then, given the split-sequence *x*, the construction of the 20 amino acids may be transformed to a 20-dimensional numeric vector *Vx*:

3 $$V_{x} = \, \left[ {P_{x} \left( 1 \right), \, P_{x} \left( 2 \right), \, \ldots , \, P_{x} \left( {20} \right) \, } \right]$$

### Feature selection from the encoded data

Both phosphorylation and non-phosphorylation fragments encoded a large number of feature variables. However, a prediction model based on a large number of features increases the computational load and creates different types of complexities. Furthermore, the features with similar abundance patterns in both positive samples (phosphorylation) and negative samples (non-phosphorylation) groups cannot increase the prediction performance. So, these types of features are usually removed from the encoded dataset to develop an effective prediction model. This study used the Kruskal–Wallis (KS)^30^ non-parametric test as a feature selection method. We selected the highest 1500 features out of 2646 CKSAAP features and 400 features out of 504 binary features by the KS statistical test to develop the prediction models.

### Learning classifier

We considered three popular classifiers (Random Forest (RF), AdaBoost (ADA) & Support Vector Machine (SVM)) for comparisons based on the encoded protein sequences to create a more effective predictor for protein phosphorylation site prediction. Let us consider a dataset consisting of *n* training data (${{\varvec{x}}}_{1}$,${y}_{1}$), (${{\varvec{x}}}_{2}$,${y}_{2}$),…, (${{\varvec{x}}}_{n}$,${y}_{n}$), where ${{\varvec{x}}}_{i}$ is an input vector in space X ⊆ ${R}^{m}$ and ${y}_{i}$ is the response variable that takes value + 1 (phosphorylation site) and − 1 (non- phosphorylation site). The main task is to classify a new sample of x windows into one of the two classes (+ 1, − 1). For the convenience of the readers, let us introduce together those classifiers as follows. Let us introduce these classifiers together as follows for the convenience of the readers.

#### Random forest (RF)

The random forest (RF) classifier is a popular statistical learning algorithm and is widely used in bioinformatics research^34,37,44,46,47,49,50^. Generally, the whole process of random forest is completed through two steps; the first is to create a random forest classifier. The second step is to predict with the help of the random forest classifier created in the first step. For better presentation, let (*X*, *Y*) = {(${{\varvec{x}}}_{1}$,${y}_{1}$), (${{\varvec{x}}}_{2}$,${y}_{2}$)… (${{\varvec{x}}}_{n}$,${y}_{n}$)}. Then B (*b* = 1, …, *B*) times selects a random sample (${X}_{b}$, ${Y}_{b}$ with replacement from the given dataset (*X,Y*) and train a regression tree ${f}_{b}$ on (${X}_{b}$, ${Y}_{b}$) to fit trees to these samples. After training, predictions for new samples ***x****’* can be written as,

4 $$\widehat{f}=\frac{1}{B}\sum_{b=1}^{B}{f}_{b}\left({x}^{^{\prime}}\right)$$

R package ‘randomForest’ was used in this paper to implement the random forest algorithm^55^.

#### AdaBoost

AdaBoost is an efficient meta-algorithm for machine learning^42^. In this paper, AdaBoost is denoted as ADA. It is efficient in the sense that subsequent weak learners are modified in favor of those instances that were misclassified by previous classifiers. It can be described as follows:

Training dataset: *{(x*_*i*_*,y*_*i*_*); i* = *1,2,…,n* }.

Suppose there are *T* weak classifiers defined by ${f}_{t}(x);t=\mathrm{1,2},3,\dots ,T$ satisfying

$${y}_{t}=sign({f}_{t}(x))=\pm 1;t=\mathrm{1,2},3,\dots ,T$$

Then the AdaBoost classifier is defined by

$${F}_{T}\left({\varvec{x}}\right)=\sum_{t=1}^{T}{\alpha }_{t}{f}_{t}\left({\varvec{x}}\right),$$

where $${\alpha }_{t}=\frac{1}{2}log\frac{1-{\varepsilon }_{t}({f}_{t})}{{\varepsilon }_{t}({f}_{t})}$$,$${\varepsilon }_{t}\left({f}_{t}\right)=\underset{f\epsilon F}{min}{\varepsilon }_{t}\left({f}_{t}\right),{f}_{t}=ar\underset{f\epsilon F}{gmin}{\varepsilon }_{t}\left(f\right), {\varepsilon }_{t}(f)=\sum_{i=1}^{n}I({y}_{i}\ne {f}_{t}({x}_{i})){w}_{t}(i)/\sum_{j=1}^{n}{w}_{t}(j)$$, $${w}_{t+1}(i)={w}_{t}(i)exp\{-{\alpha }_{t}{f}_{t}({x}_{i}){y}_{i}\}$$

Then the classification rule is defined as

5 $${f}_{T}\left(x\right)=sign\left({F}_{T}\left(x\right)\right)=\pm 1,$$

R package 'ada' was used in this paper to implement the AdaBoost algorithm^42^.

#### Support vector machine (SVM)

The purpose of SVM is to identify a hyperplane in an m-dimensional space that specifically classifies the data points^42,43,47^. Let us consider that the data points consist of *n* training data (${{\varvec{x}}}_{1}$,${y}_{1}$), (${{\varvec{x}}}_{2}$,${y}_{2}$)… (${{\varvec{x}}}_{n}$,${y}_{n}$), where ${x}_{i}$ is an input vector in the space X ⊆ ${R}^{m}$ and ${y}_{i}$ is the output variable that takes values 1 for succinylated site and -1 for non-succinylated site. A hyperplane in high dimensional space is constructed by the SVM approach, which can be used in both classification and regression. The hyperplane may be written in the following form:

6 $${W}^{T}X+b=0$$

where b is scalar, and W is a normalized m-dimensional vector perpendicular to the divided hyperplane. If the data can be separated in a linear way, then the two classes can be written as follows: ${W}^{T}X+b>0\ if\ {y}_{i}=1$ and ${W}^{T}X+b<0\ if\ {y}_{i}=-1$. If the data is not linearly separable, then SVM uses kernel functions to transform the original data to a reasonable space, with high dimensional space that can separate the classes in phosphorylation and non-phosphorylation site. In such a situation, the hyperplane can be written as follow:

7 $$f\left({\varvec{x}}\right)= {\sum }_{i=1}^{m}{\alpha }_{i}{y}_{i} K \left({{\varvec{x}}}_{i},{\varvec{x}}\right)+b$$

where, ${\alpha }_{n}$ is Lagrange multiplier, ${y}_{i}$ is the class label that belongs to (− 1, 1), and $K{({\varvec{x}}}_{i},{\varvec{x}})$ is the Kernel function between ${x}_{i}$ and $x$. In this study, we have adopted the kernel as a radial basis function (RBF). R package 'e1071’ was used in this paper to implement the SVM algorithm^35^.

### Fusion model

To increase the efficiency of their prediction models, many authors used fusion techniques^46,56,57^.

We have attempted to boost the efficiency of our prediction model in this article by combining binary, CKSAAP, and AAC encoding schemes with the RF classifier as follows,

8 $${\text{RF}}\left( {{\text{CKSAAP}},{\text{ Binary}},{\text{ AAC}}} \right) \, = w_{1} \times {\text{RF}}\left( {{\text{CKSAAP}}} \right) \, + w_{2} \times {\text{RF}}\left( {{\text{Binary}}} \right) \, + w_{3} \times {\text{RF}}\left( {{\text{AAC}}} \right)_{ }$$

where RF(CKSAAP), RF(Binary), and RF(AAC) denote the RF classification scores estimated with CKSAAP, binary, and AAC encoding schemes, respectively. The values of *w1*, *w2, and w*_*3*_ were selected based on the ratio of individual prediction performance of RF(Binary), RF(CKSAAP), and RF(AAC) satisfying *w*_*1*_ + *w*_*2*_ + *w*_*3*_ = 1. To compare the performance of the RF based prediction model with the performance of ADA and SVM based prediction models, we also enhanced the prediction performance of the ADA and SVM based prediction model by combining binary, CKSAAP, and AAC encoding methods.

### Performance evaluation measures

In the present study, some widely used performance measures including true positive rate (TPR) known as ‘sensitivity’, true negative rate (TNR) known as ‘specificity’, false negative rate (FNR), accuracy (ACC), misclassification rate(MCC), Mathew correlation coefficient (MCC), receiving operating characteristics (ROC) curve, area under the ROC curve (AUC) and partial AUC (pAUC) were considered to select the best prediction model. These measurement scores are calculated as

9 $$TPR=\frac{n(TP)}{n(TP)+n(FN)};0\le TPR\le 1$$

10 $$\mathrm{FPR}=\frac{n\left(FP\right)}{n\left(TN\right)+n\left(FP\right)}; 0\le FPR\le 1$$

11 $$\mathrm{TNR}= \frac{n(TN)}{n(TN)+n(FP)}; 0\le TNR\le 1$$

12 $$\mathrm{FNR}=\frac{n(FN)}{n(TP)+n(FN)}; 0\le FNR\le 1$$

13 $$ACC=\frac{n(TP)+n(TN)}{n(TP)+n(FP)+n(TN)+n(FN)}; 0\le ACC\le 1$$

14 $$MCR=\frac{n(FP)+n(FN)}{\begin{array}{c}n(TP)+n(FP)+n(TN)+n\left(FN\right)\\ \end{array}}; 0\le MCR\le 1$$

15 $$MCC=\frac{\left(n(TP)\times n(TN\right))-(n\left(FP\right)\times n\left(FN\right))}{\sqrt{(n\left(TP\right)+n\left(FN\right))\times (n\left(TN\right)+n\left(FP\right))\times (n\left(TP\right)+n\left(FP\right))\times (n(TN)+n(FN))}};-1\le MCC\le 1$$

where *n*(*TN*): True Negative number, *n(TP)*: True Positive number, *n(FN)*: False Negative number, *n(FP)*: False Positive number. The ROC curve is formed by plotting TPR: sensitivity against FPR = (1-specificity). Obviously TPR + FNR = 1, TNR + FPR = 1, (FPR, FNR) → (0, 0) implies MCR → 0 and (TPR, TNR, ACC, MCC, AUC) → (1, 1, 1, 1, 1), conversely (FPR, FNR) → (1, 1) implies MCR → 1 and (TPR, TNR, ACC, MCC, AUC) → (0, 0, 0, 0, 0, − 1). Therefore, a prediction model that produces comparatively larger values of TPR, TNR, ACC, MCC, and AUC, and the smaller values of FPR, FNR, and MCR, indicates the better prediction model.

#### *K*-fold cross-validation (CV)

To perform *K*-fold CV, the dataset “D” was randomly partitioned into *k* = 5 disjoint subsets (*D1, D2*,…, *Dk*) such that every subset contains almost equal elements. The (*K-1*) subsets were used as train the prediction model and the remaining one set was used to validate the prediction model by computing different performance scores with measures *TPR/SN*, *TNR/SF*, *FPR*, *FNR*, *MCR*, *ACC*, *MCC *& *AUC*. This procedure was replicated K = 5 times by changing the validation set with one of the training sets. Then the average score for each performance measure was computed to evaluate the prediction model.

## Results

To develop an effective model for prediction of serine phosphorylation site mapping on *SP*, we considered the dataset that was consisted of 766 serine phosphorylated protein sequences with 4530 positive sites and 36,705 negative sites. The redundant sequences were removed from this dataset by using the CD-HIT cut-off at 30%. Then we created the training dataset by randomly taking 690 (90%) phosphorylated protein sequences with 3925 positive and 33,360 negative window sites. The rest 76 (10%) phosphorylated protein sequences with 605 positive and 3345 negative window sites were considered to create the independent test set. Then we selected WS = 25 by two sample logo (TSL) analysis to generate the effective feature variables for both training and independent test datasets. Each window was represented by a 25 (± 12)-residue peptide segment with *S* in the middle (see “The TSL analysis”). However, the total number of positive windows (*n*_*1*_ = 3925) and negative windows (*n*_*2*_ = 33,360) were clearly unbalanced in the training dataset. Therefore, we created 3 comparatively balanced datasets with 1:1, 1:2, and 1:3 ratios of positive and negative window samples, respectively, to select one of them for developing a better predictor as discussed in section “Data preparation and overview on the development of the proposed prediction model”. We compared the training performance of different prediction models in section “Performance of prediction models with the training dataset”. Then we evaluated their performances by 5-fold CV in section “Prediction performance evaluation by 5-fold cross validation (CV)”. The success ratings based on the independent test dataset were addressed in section “Performance of prediction models with the independent test dataset”.

### The TSL analysis

**Figure 2:**
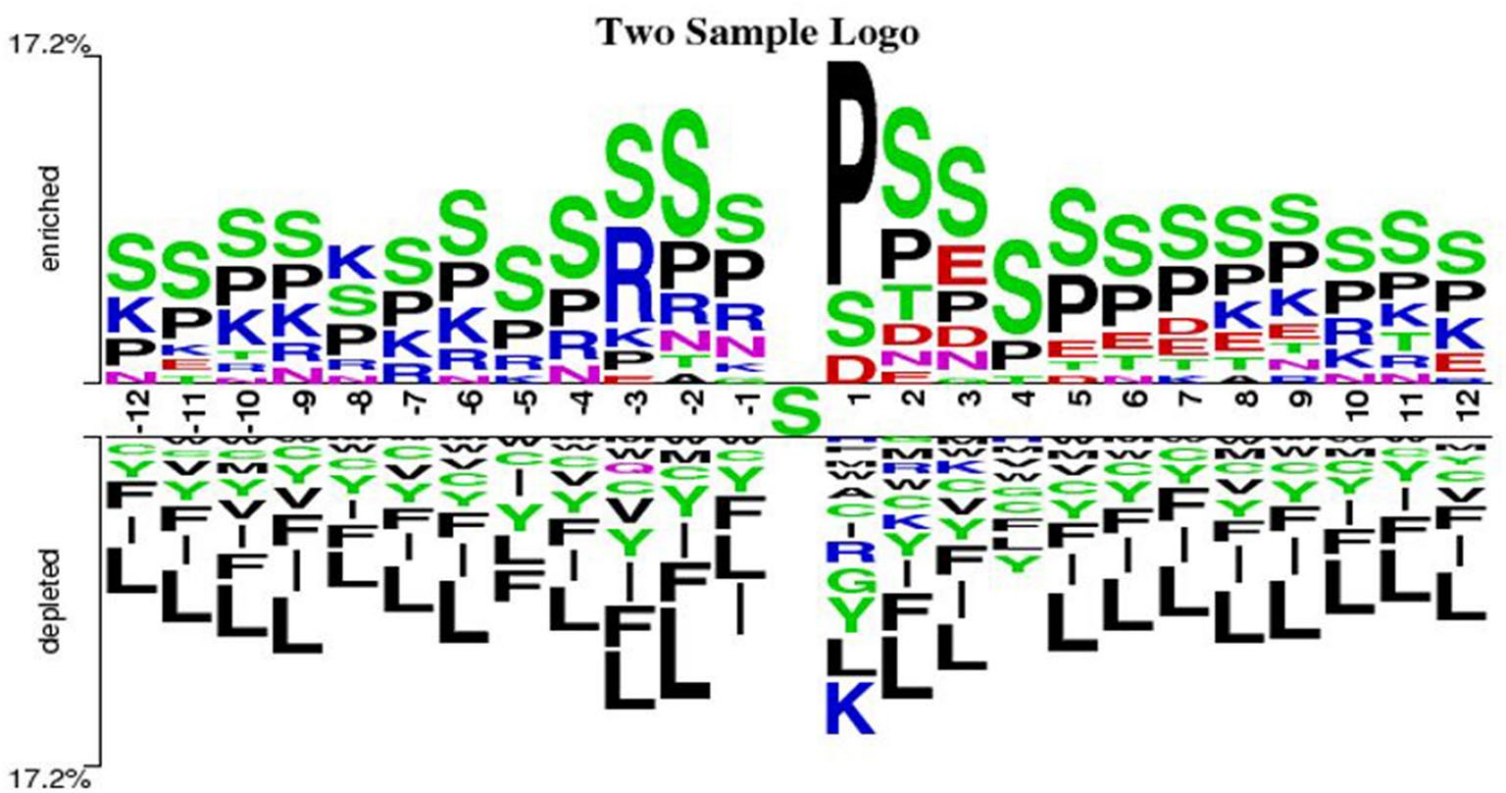
Two study logos program^58^ presents the occurrences of amino acid propensities of surrounding positive windows (phosphorylation site) and negative windows (non-phosphorylation sites) of size 25.

To investigate the adequacy of the dataset for the development of prediction model, we conducted two sample logo (TSL) tests. The neighboring phosphorylation and non-phosphorylation sites for the training dataset are shown in Fig. 2 through TSL software^57^. Positive or negative samples, respectively, define residues at each position above and below the X-axis. The height of the letter accommodating the corresponding residue was shown in proportion to the percentage of over-represented (if positive) or underrepresented samples (if negative). The total percentage of these positive/negative residues is represented by the y-axis. The amino acid occurrences between positive and negative phosphorylation protein samples are described by TSL logos. Figure 2 shows the TSL of 25-mer (− 12, + 12)WS. It represented some significantly enriched (over represented) or depleted (under represented) residues with the flanking of focused phosphorylation sites (p-value < 0.05), which indicates that the dataset is adequate to develop a prediction model with WS = 25. Similarly, Supplementary Figs. S1 and S2 showed that the dataset is also suitable for WS = 21 and 27 to develop the prediction model.

### Performance of prediction models with the training dataset

At first, we trained ADA based 7 prediction models denoted as ADA(CKSAAP), ADA(Binary), ADA(AAC), ADA(CKSAAP, Binary), ADA(CKSAAP, AAC), ADA(Binary, AAC), and ADA(CKSAAP, Binary, AAC) by using the training dataset that contained 1:2 ratio of positive and negative samples. Similarly, 7 prediction models based on each of SVM and RF classifiers were trained by the same dataset. We computed different performance scores (TPR, TNR, FNR, MCR, ACC, MCC & AUC) at FPR = 0.20 to investigate the success for each of those 21 prediction models (see Table 1).

**Table 1:** Training performance scores at FPR = 0.20 for 21 prediction models that were trained by 1:2 ratio of positive and negative samples.

Predictors	TPR	TNR	FNR	ACC	MCC	MCR	AUC	pAUC
ADA (CKSAAP)	0.763	0.801	0.237	0.877	0.662	0.172	0.891	0.121
ADA (binary)	0.757	0.800	0.243	0.863	0.657	0.210	0.872	0.119
ADA (AAC)	0.750	0.802	0.250	0.862	0.643	0.198	0.876	0.115
ADA (CKSAAP, binary)	0.772	0.802	0.220	0.907	0.645	0.142	0.923	0.141
ADA (CKSAAP, AAC)	0.757	0.801	0.243	0.868	0.656	0.189	0.887	0.133
ADA (binary, AAC)	0.761	0.800	0.239	0.869	0.658	0.186	0.899	0.139
**ADA (CKSAAP, binary, AAC)**	**0.789**	**0.801**	**0.210**	**0.912**	**0.720**	**0.121**	**0.933**	**0.154**
SVM (CKSAAP)	0.769	0.801	0.233	0.887	0.668	0.173	0.899	0.132
SVM (binary)	0.765	0.800	0.221	0.879	0.668	0.167	0.898	0.120
SVM (AAC)	0.638	0.801	0.362	0.737	0.541	0.268	0.820	0.079
SVM (CKSAAP, binary)	0.869	0.801	0.121	0.939	0.787	0.100	0.942	0.131
SVM (CKSAAP, AAC)	0.668	0.802	0.332	0.779	0.575	0.243	0.848	0.071
SVM (binary, AAC)	0.675	0.801	0.325	0.781	0.578	0.241	0.850	0.072
**SVM (CKSAAP, binary, AAC)**	**0.878**	**0.801**	**0.121**	**0.946**	**0.797**	**0.09**	**0.952**	**0.143**
RF (CKSAAP)	0.867	0.801	0.113	0.935	0.789	0.107	0.934	0.152
RF (binary)	0.854	0.800	0.123	0.939	0.781	0.109	0.927	0.145
RF (AAC)	0.761	0.802	0.239	0.905	0.627	0.159	0.913	0.129
RF (CKSAAP, binary)	0.888	0.802	0.111	0.945	0.789	0.100	0.965	0.198
RF (CKSAAP, AAC)	0.857	0.801	0.143	0.932	0.778	0.113	0.942	0.157
RF (binary, AAC)	0.859	0.801	0.141	0.934	0.779	0.110	0.947	0.161
**RF (CKSAAP, binary, AAC)**	**0.898**	**0.802**	**0.121**	**0.957**	**0.792**	**0.060**	**0.977**	**0.197**

Better results with each of ADA, SVM and RF were highlighted by bold values.

At first, we investigated the performance of ADA based 7 prediction models. We observed that 3 encodings (Binary, CKSAAP, AAC) based fusion model produces largest scores of TPR (0.789), TNR (0.801), ACC (0.912), MCC (0.720), AUC (0.933) and pAUC (0.154), and smallest scores of FNR (0.210) and MCR (0.121). Thus ADA based fusion prediction model with 3 encoding features showed better performance compared to the other 6 ADA based prediction modes. Similarly, each of SVM and RF based prediction models with the fusing of those 3 types of encoding features, also showed better performance compared to their 6 alternative prediction modes. Then we compared the ADA, SVM, and RF based three best prediction models and observed that the RF-based fusion model RF(Binary, CKSAAP, AAC) produces the larger TPR (0.898), TNR (0.802), ACC (0.957), MCC (0.792), AUC (0.977) and pAUC (0.199), and the smaller FNR (0.121) and MCR (0.06) compare to the ADA and SVM based best prediction models that were denoted as ADA (Binary, CKSAAP, AAC) and SVM (Binary, CKSAAP, AAC). That is, the proposed RF based fusion prediction model RF (Binary, CKSAAP, AAC) outperforms the other 20 prediction models as discussed above with the training dataset corresponding to 1:2 ratio of positive and negative samples. Similarly, it showed better performance compare to both ADA and SVM based fusion models with the training datasets corresponding to 1:1 and 1:3 ratio cases also (see Tables S1 and S2 in the Supplementary File S1). Again, we observed from Tables 1, S1 and S2 that the proposed RF based fusion prediction model RF (Binary, CKSAAP, AAC) shows slightly better performance for both 1:2 and 1:3 ratio cases compared to the 1:1 ratio of positive and negative samples. It was also observed that its performance is almost same for both 1:2 and 1:3 ratio cases.

### Prediction performance evaluation by 5-fold cross validation (CV)

**Figure 3:**
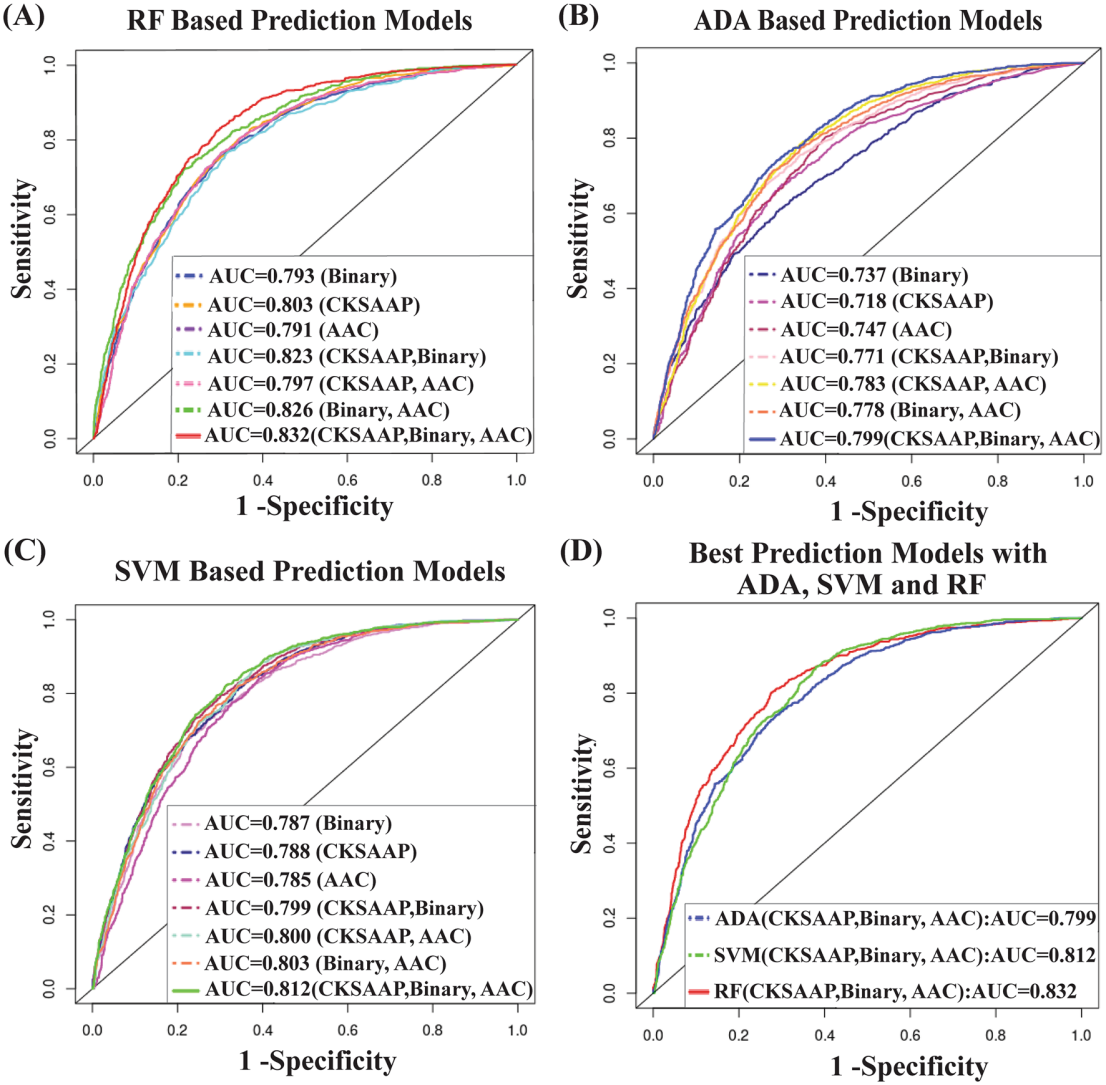
Performance of 21 prediction models by 5-fold CV results based on the training dataset that was consisted of 1:2 ratio of positive and negative samples. (**A**) ROC curves with the RF based 7 different prediction models, (**B**) ROC curves with the ADA based 7 different prediction models, (**C**) ROC curves with the SVM based 7 different prediction models, and (**D**) ROC curves for the best prediction models with ADA, SVM, and RF.

To evaluate the prediction performance of the proposed RF based fusion prediction model RF (Binary, CKSAAP, AAC) compare to other 20 candidate prediction models by 5-fold CV, the training dataset corresponding to 1:2 ratio of 3925 positive and 7850 negative window samples was partitioned into 5 mutually exclusive subgroups (G1, G2, G3, G4, G5) such that each subgroup consists of 1:2 ratio of positive and negative samples. Each subgroup obviously consisted of around 785 positive window samples (5% of 3925) and around 1570 negative window samples (5% of 7850). Within 5 replications, the 5-fold CV was completed. A phase-1, four subgroups (G2, G3, G4 & G5) that contains 80% samples of the training dataset, including the 1:2 ratio of positive and negative cases, were used to train all 21 prediction models. The other group G1 was utilized to validate the trained models by computing TPR, TNR, FNR, ACC, MCR, MCC, ROC, AUC, and pAUC. At phase 2, four subgroups (G1, G3, G4 & G5) containing approximately 80% window samples were also used as before to train all prediction models. The other G2 group was used to compute the performance indicators (TPR, TNR, FNR, ACC, MCR, MCC, ROC, AUC, and pAUC) as before. In this case, only the subgroup pair G1–G2 interchanged their positions between the training and test sets. Similarly, subgroup pairs G2–G3, G3–G4, & G4–G5 exchanged, their positions between the training and test sets, respectively, at another three (3) loops. The performance scores of TPR, TNR, FNR, ACC, MCR, MCC, and pAUC were calculated by fixing the cutoff point at FPR = 0.20 for each of 5 loops. Then we computed the average performance scores of TPR, TNR, FNR, ACC, MCR, MCC, ROC, AUC, and pAUC and displayed the summary results in Table 2. The values in the first bracket represent the standard error (SE) of performance scores. As before, at first, we investigated the performance of ADA based 7 prediction models. We observed that 3 encodings (Binary, CKSAAP, AAC) based fusion model produces largest average scores of TPR (0.654), TNR (0.801), ACC (0.721), MCC (0.456), AUC (0.799) and pAUC (0.1404), and smallest scores of FNR (0.346) and MCR (0.287). Thus ADA based fusion prediction model with 3 encoding schemes showed better performance compare to the other 6 ADA based prediction modes (see Table 2 and Fig. 3A). Similarly, SVM and RF based fusion prediction models with 3 encoding schemes also showed better performance compared to their 6 alternative prediction modes (see Table 2 and Fig. 3B,C). Then we compared the ADA, SVM, and RF based three best prediction models and observed that the fusion model RF(Binary, CKSAAP, AAC) produces the larger TPR (0.810), TNR (0.802), ACC (0.778), MCC (0.666), AUC (0.832) and pAUC (0.168) and the smaller FNR (0.190) and MCR (0141) compare to the ADA and SVM based best prediction models that were written as ADA(Binary, CKSAAP, AAC) and SVM(Binary, CKSAAP, AAC) as before (see Table 2 and Fig. 3D). That is, the proposed RF based prediction model RF (Binary, CKSAAP, AAC) performed much better compared to the other 20 prediction models by 5-fold CV with the training dataset corresponding to 1:2 ratio of positive and negative samples. Similarly, it showed better performance compared to both ADA and SVM based fusion models by 5-fold CV with the training datasets corresponding to 1:1 and 1:3 ratio cases also (see Figs. S3A and S4A in the Supplementary File S2). Again, we observed from Figs. 3D, S3A, and S4A that the proposed RF based fusion prediction model RF (Binary, CKSAAP, AAC) show slightly better performance for both 1:2 and 1:3 ratio cases compared to the 1:1 ratio of positive and negative samples. It was also observed that its performance is almost same for both 1:2 and 1:3 ratio cases. Thus, the RF-based fussing model with 3 encoding schemes (Binary, CKSAAP, AAC) showed better performance compared to the ADA and SVM based best prediction models by 5-fold CV also.

**Table 2:** Performance scores at FPR = 0.20 for 21 prediction models by 5-fold CV with the training dataset that was consisted of 1:2 ratio of positive and negative samples.

Predictors classifier (encoding)	TPR	TNR	FNR	ACC	MCC	MCR	AUC	pAUC
ADA (CKSAAP)	0.676 (0.32)	0.800 (0.00)	0.323 (0.32)	0.689 (0.01)	0.378 (0.16)	0.311 (0.01)	0.737 (0.04)	0.12 (0.06)
ADA (binary)	0.613 (0.03)	0.800 (0.01)	0.386 (0.03)	0.657 (0.01)	0.315 (0.03)	0.343 (0.01)	0.718 (0.03)	0.11 (0.07)
ADA (AAC)	0.644 (0.31)	0.801 (0.00)	0.355 (0.31)	0.692 (0.03)	0.383 (0.02)	0.291 (0.09)	0.747 (0.05)	0.133 (0.04)
ADA (CKSAAP, binary)	0.650 (0.24)	0.800 (0.01)	0.349 (0.24)	0.702 (0.12)	0.407 (0.24)	0.297 (0.12)	0.771 (0.10)	0.136 (0.05)
ADA (CKSAAP, AAC)	0.661 (0.09)	0.800 (0.00)	0.339 (0.09)	0.712 (0.10)	0.417 (0.21)	0.289 (0.10)	0.783 (0.09)	0.139 (0.03)
ADA (binary, AAC)	0.653 (0.12)	0.800 (0.00)	0.347 (0.12)	0.710 (0.13)	0.412 (0.11)	0.292 (0.13)	0.778 (0.10)	0.137 (0.09)
**ADA (CKSAAP, binary, AAC)**	**0.654** **(0.08)**	**0.801** **(0.00)**	**0.346** **(0.08)**	**0.721** **(0.01)**	**0.456** **(0.15)**	**0.287** **(0.07)**	**0.799** **(0.02)**	**0.140** **(0.06)**
SVM (CKSAAP)	0.677 (0.16)	0.800 (0.00)	0.323 (0.03)	0.712 (0.12)	0.425 (0.07)	0.287 (0.02)	0.788 (0.06)	0.143 (0.07)
SVM (binary)	0.683 (0.02)	0.800 (0.00)	0.317 (0.03)	0.718 (0.01)	0.438 (0.15)	0.281 (0.09)	0.787 (0.08)	0.138 (0.04)
SVM (AAC)	0.681 (0.12)	0.801 (0.00)	0.316 (0.12)	0.704 (0.13)	0.382 (0.06)	0.325 (0.01)	0.785 (0.03)	0.134 (0.05)
SVM (CKSAAP, binary)	0.711 (0.08)	0.800 (0.00)	0.293 (0.29)	0.728 (0.11)	0.445 (0.26)	0.256 (0.11)	0.799 (0.23)	0.146 (0.09)
SVM (CKSAAP, AAC)	0.543 (0.13)	0.802 (0.00)	0.456 (0.09)	0.667 (0.23)	0.376 (0.12)	0.356 (0.04)	0.800 (0.03)	0.154 (0.11)
SVM (binary, AAC)	0.567 (0.12)	0.801 (0.00)	0.432 (0.11)	0.684 (0.13)	0.382 (0.21)	0.324 (0.12)	0.803 (0.05)	0.169 (0.10)
**SVM (CKSAAP, binary, AAC)**	**0.598** **(0.08)**	**0.802** **(0.00)**	**0.401** **(0.13)**	**0.700** **(0.12)**	**0.422** **(0.09)**	**0.312** **(0.02)**	**0.812** **(0.07)**	**0.170** **(0.08)**
RF (CKSAAP)	0.798 (0.15)	0.800 (0.00)	0.201 (0.15)	0.749 (0.26)	0.500 (0.20)	0.251 (0.11)	0.803 (0.16)	0.145 (0.08)
RF (binary)	0.735 (0.09)	0.800 (0.00)	0.264 (0.09)	0.721 (0.14)	0.443 (0.01)	0.278 (0.02)	0.793 (0.13)	0.143 (0.06)
RF (AAC)	0.691 (0.15)	0.801 (0.00)	0.308 (0.15)	0.786 (0.26)	0.584 (0.20)	0.213 (0.11)	0.791 (0.16)	0.141 (0.08)
RF (CKSAAP, binary)	0.806 (0.02)	0.800 (0.00)	0.193 (0.01)	0.754 (0.02)	0.510 (0.06)	0.246 (0.10)	0.823 (0.03)	0.158 (0.02)
RF (CKSAAP, AAC)	0.681 (0.13)	0.800 (0.00)	0.319 (0.13)	0.659 (0.23)	0.502 (0.18)	0.182 (0.09)	0.797 (0.14)	0.151 (0.08)
RF (binary, AAC)	0.725 (0.09)	0.802 (0.00)	0.275 (0.09)	0.671 (0.14)	0.588 (0.01)	0.185 (0.02)	0.826 (0.13)	0.159 (0.06)
**RF (CKSAAP, binary, AAC)**	**0.810** **(0.02)**	**0.802** **(0.00)**	**0.190** **(0.01)**	**0.778** **(0.02)**	**0.666** **(0.06)**	**0.141** **(0.10)**	**0.832** **(0.03)**	**0.168** **(0.02)**

Better results with each of ADA, SVM and RF were highlighted by bold values.

The values within the first bracket indicate the standard error (SE).

### Performance of prediction models with the independent test dataset

To evaluate the independent test performance of the proposed prediction model compared to the other 20 candidate models, all candidate models were trained by the training dataset of 1:2 ratio of 3925 positive and 7850 negative window samples, as mentioned earlier in sections “Data preparation and overview on the development of the proposed prediction model” and “Performance of prediction models with the training dataset”. The independent test dataset consisted of 76 proteins with 982 positive samples and 1964 negative samples, as introduced in section “Data preparation and overview on the development of the proposed prediction model”. Then we computed the performance scores TPR, TNR, FNR, ACC, MCR, MCC, ROC, AUC, and pAUC as before based on the independent test dataset.

**Figure 4:**
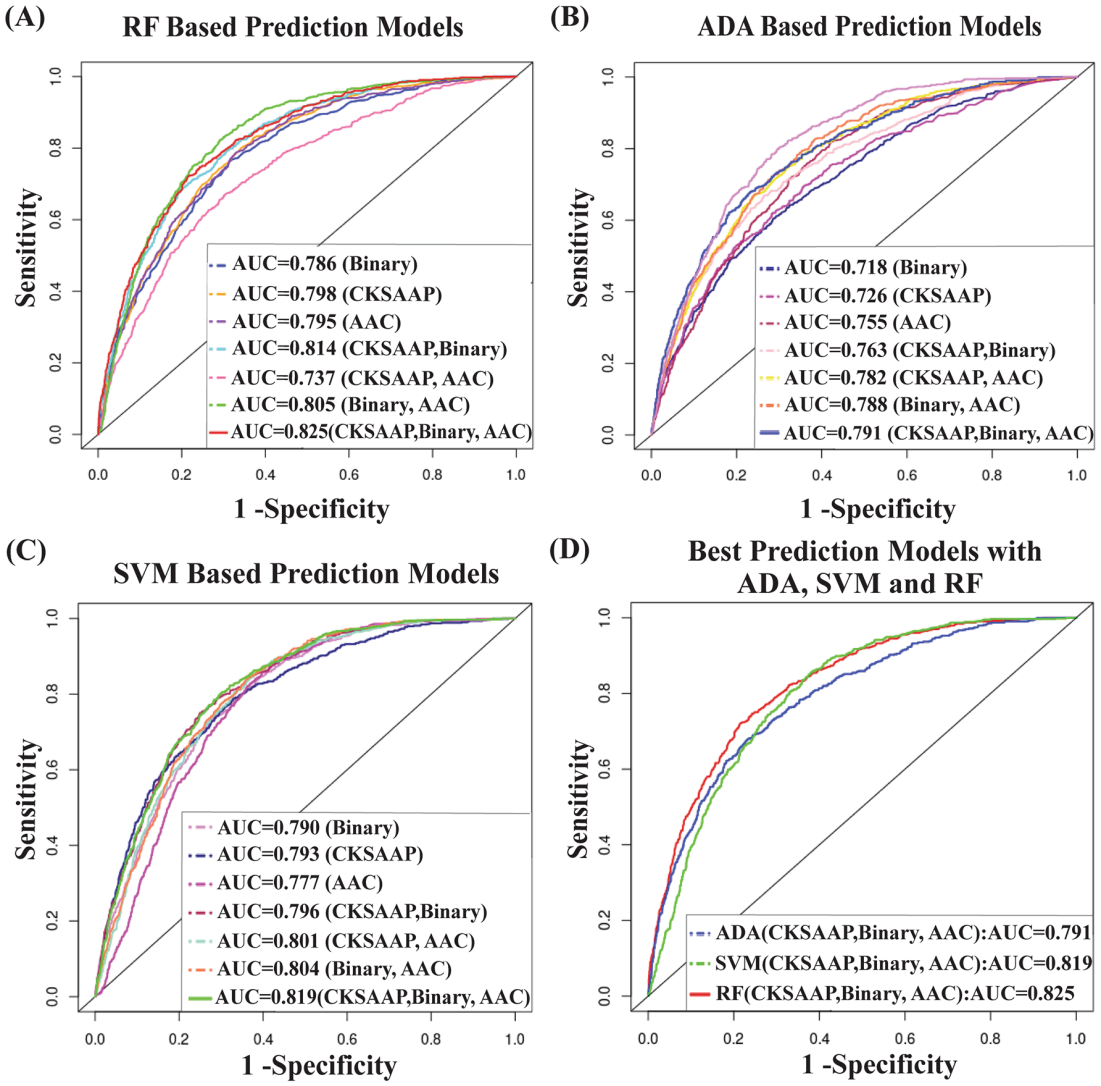
Independent test performance for 21 different candidate prediction models. (**A**) ROC curves with the RF based 7 different prediction models, (**B**) ROC curves with the ADA based 7 different prediction models, (**C**) ROC curves with the SVM based 7 different prediction models, and (**D**) ROC curves for the best prediction models with ADA, SVM, and RF.

At first, we assess the performance of ADA based 7 prediction models as before. We found that 3 encodings (Binary, CKSAAP, AAC) based fusion model produces highest average scores of TPR (0.639), TNR (0.801), ACC (0.700), MCC (0.502), AUC (0.791) and pAUC (0.146), and lowest scores of FNR (0.381) and MCR (0.299). Thus ADA based fusion prediction model with 3 encoding features showed better performance compare to the other 6 ADA based prediction modes (see Table 3 and Fig. 4A). Similarly, SVM and RF based fusion prediction models with 3 encoding schemes also showed better performance compared to their 6 alternative prediction modes (see Table 3 and Figs. 4B,C). Then we compared the ADA, SVM, and RF based three best prediction models and observed that the fusion model RF(Binary, CKSAAP, AAC) produces the larger TPR (0.798), TNR (0.802), ACC (0.791), MCC (0.629), AUC (0.825) and pAUC (0.169) and the smaller FNR (0.201) and MCR (0145) compare to the ADA and SVM based best prediction models that were written as ADA (Binary, CKSAAP, AAC) and SVM (Binary, CKSAAP, AAC) as before (see Table 3 and Fig. 4D). That is, the proposed RF based prediction model RF(Binary, CKSAAP, AAC) showed much better independent test performance compared to the other 20 candidate prediction models with the training dataset corresponding to 1:2 ratio of positive and negative samples. Similarly, it showed better independent test performance compared to both ADA and SVM based fusion models with the training datasets corresponding to 1:1 and 1:3 ratio cases also (see Figs. S3B and S4) in the Supplementary file S2). Again, we observed from Figs. 4D, S3B, and S4B that the proposed RF based fusion prediction model RF(Binary, CKSAAP, AAC) shows slightly better performance for both 1:2 and 1:3 ratio cases compared to the 1:1 ratio case. It was also observed that its performance is almost same for both 1:2 and 1:3 ratio cases. Thus, the RF-based fussing model with 3 encoding schemes (Binary, CKSAAP, AAC) showed better performance compared to the ADA and SVM based best prediction models with the independent test dataset.

**Table 3:** Independent test performance scores at FPR = 0.20 for 21 prediction models that were trained by 1:2 ratio of positive and negative samples.

Predictors	TPR	TNR	FNR	ACC	MCC	MCR	AUC	pAUC
ADA (CKSAAP)	0.631	0.800	0.369	0.665	0.331	0.334	0.726	0.121
ADA (binary)	0.614	0.800	0.385	0.657	0.316	0.342	0.718	0.118
ADA (AAC)	0.618	0.802	0.381	0.669	0.426	0.338	0.755	0.122
ADA (CKSAAP, binary)	0.635	0.800	0.364	0.697	0.397	0.303	0.763	0.138
ADA (CKSAAP, AAC)	0.621	0.801	0.398	0.682	0.467	0.309	0.782	0.140
ADA (binary, AAC)	0.626	0.802	0.393	0.691	0.487	0.308	0.788	0.142
**ADA (CKSAAP, binary, AAC)**	**0.639**	**0.801**	**0.381**	**0.700**	**0.502**	**0.299**	**0.791**	**0.146**
SVM (CKSAAP)	0.718	0.800	0.281	0.721	0.442	0.278	0.793	0.137
SVM (binary)	0.677	0.800	0.322	0.716	0.433	0.283	0.790	0.136
SVM (AAC)	0.645	0.901	0.345	0.772	0.563	0.227	0.777	0.122
SVM (CKSAAP, binary)	0.728	0.800	0.278	0.734	0.467	0.268	0.796	0.140
SVM (CKSAAP, AAC)	0.698	0.902	0.301	0.761	0.526	0.238	0.801	0.145
SVM (binary, AAC)	0.701	0.901	0.298	0.812	0.667	0.209	0.804	0.149
**SVM (CKSAAP, binary, AAC)**	**0.703**	**0.902**	**0.297**	**0.811**	**0.666**	**0.208**	**0.819**	**0.151**
RF (CKSAAP)	0.772	0.800	0.227	0.739	0.479	0.261	0.798	0.143
RF (binary)	0.729	0.800	0.271	0.716	0.432	0.283	0.786	0.142
RF (AAC)	0.732	0.902	0.267	0.771	0.544	0.228	0.795	0.147
RF (CKSAAP, binary)	0.777	0.800	0.222	0.749	0.478	0.261	0.814	0.154
RF (CKSAAP, AAC)	0.732	0.802	0.267	0.671	0.544	0.228	0.737	0.124
RF (binary, AAC)	0.761	0.802	0.239	0.760	0.627	0.159	0.805	0.129
RF (CKSAAP, binary, AAC)	**0.798**	**0.802**	**0.201**	**0.791**	**0.629**	**0.145**	0.825	**0.169**

Better results with each of ADA, SVM and RF were highlighted by bold values.

## Discussion

In this study, we proposed an effective computational model for prediction of serine phosphorylation sites mapping on *SP* by fusion three encoding schemes (CKSAAP, Binary, AAC) with RF classifier based on 1:2 ratio of positive and negative window samples with respect to the cutoff value of CD-HIT at 30% and window size (WS) at 25. We selected this model as the best prediction model compare to the SVM and ADA based prediction models, giving the weight on its largest performance scores with SN, SP, ACC, MCC, and AUC, and the smallest performance scores with FPR, FNR and MCR. We observed from our investigation that all candidate prediction models show slightly better performance with both 1:2 and 1:3 ratio cases compared to the 1:1 ratio of positive and negative window samples (see Tables 1, S1 and S2, and Figs. 3, 4, S3 and S4). It was also observed that their performance was almost same with 1:2 and 1:3 ratios of positive and negative samples. The negative samples were representative for all of 3 ratio cases, since negative samples were selected randomly in each ratio case. Moreover, better estimates of the model parameters not only depend on representative samples but also on the larger sample size. Therefore, we selected the dataset corresponding to the 1:2 ratios of positive and negative window samples to build the prediction model, since prediction performance was almost same for both 1:2 and 1:3 ratio cases. We also observed that the prediction performances are almost same for all three window sizes at 21, 25, and 27 (See Figs. 4 and S5), which is also supported by the TSL analysis results (see Figs. 2, S1 and S2). Now, let us discuss, how we selected the RF based prediction model as the best prediction model compare to the SVM and ADA based models. To observe the training performance of the proposed prediction model in a comparison of the other candidate predictors, we computed different performance scores with the training dataset. Then we investigated their comparative performance by 5-fold CV with the training dataset. To investigate the independent test performance of the prediction models, we computed different performance scores with the independent test dataset also. In almost all cases, we observed that CKSAAP encoding feature based prediction model with each of ADA, SVM, and RF, shows slightly better performance compared to the binary and AAC encoding feature based prediction models, individually (See Tables 1, 2, 3, S1–S3, Figs. 3,4). So we provided more weight to the CKSAAP encoding compared to the binary and AAC encoding to develop the fusion model [see Eq. (8)]. Then, we observed that the training performance of the proposed RF based fusion prediction model RF (CKSAAP, Binary, AAC) are much better compared to the other 20 candidate prediction models that were denoted as ADA (CKSAAP), ADA (Binary), ADA (AAC), ADA (CKSAAP, AAC), ADA (CKSAAP, Binary), ADA (Binary, AAC), ADA (CKSAAP, Binary, AAC), SVM (CKSAAP), SVM (Binary), SVM (AAC), SVM (CKSAAP, AAC), SVM (CKSAAP, Binary), SVM (Binary, AAC), SVM (CKSAAP, Binary, AAC), RF (CKSAAP), RF (Binary), RF (AAC), RF (CKSAAP, AAC), RF (CKSAAP, Binary) and RF (Binary, AAC) (see Tables 1 and S1). Similarly, Tables 2, S2, and Fig. 3, as discussed in section “Prediction performance evaluation by 5-fold cross validation (CV)”, indicate that the proposed prediction model performs much better compared to the other 20 candidate prediction models in the case of fivefold CV. Finally, we investigated the independent test performance of the proposed prediction model based on independent test dataset and found much better performance compared to the other 20 candidate prediction models (see Tables 3, S3, and Fig. 4). Thus, we observed that the proposed RF based fusion prediction model outperforms the SVM and ADA based fusion models.

## Conclusions

Based on the protein sequence information, we developed an effective predictor to predict the serine phosphorylation sites mapping on *SP* by combining three encoding schemes, CKSAAP, binary, and AAC, with the RF classifier. We conducted a comparative study to select the better model for prediction of serine phosphorylation sites by using the experimentally detected phosphorylated protein sequences of *SP*. The 5-fold CV and independent test investigational findings indicated that our proposed approach can be more reliable to detect the phosphorylated protein compare to the other candidate prediction models. Thus, in the case of *SP* PTMs, the suggested approach can be a helpful and motivating computational resource for the prediction of serine phosphorylation sites. Finally, a user-friendly web server was developed for its implementation, which is freely accessible at http://mollah-bioinformaticslab-stat.ru.ac.bd/PredSPS/.

## Supplementary Information

Supplementary Information 1.

Supplementary Information 2.
