# Supplementary material for: Prediction of serine phosphorylation sites mapping on Schizosaccharomyces Pombe by fusing three encoding schemes with the random forest classifier
Source: Sci Rep. 2022 Feb 16;12:2632. doi: 10.1038/s41598-022-06529-5 (PMC8850546; doi:10.1038/s41598-022-06529-5)
Supplement: Supplementary file 1 — Supplementary Information 1. [file 41598_2022_6529_MOESM1_ESM.pdf]

Supplementary File 1 (File S1)

Table S1: Training performance scores at FPR=0.20 for 21 prediction models that were trained with the 1:1 ratio of positive and negative samples.

Predictors	TPR	TNR	FNR	ACC	MCC	MCR	AUC	pAUC
ADA(CKSAAP)	0.752	0.801	0.248	0.857	0.662	0.183	0.881	0.121
ADA(Binary)	0.746	0.800	0.234	0.853	0.657	0.221	0.862	0.113
ADA(AAC)	0.740	0.802	0.260	0.852	0.643	0.187	0.866	0.111
ADA(CKSAAP, Binary)	0.762	0.802	0.230	0.901	0.645	0.151	0.921	0.140
ADA(CKSAAP, AAC)	0.746	0.801	0.254	0.870	0.656	0.190	0.877	0.130
ADA(Binary, AAC)	0.750	0.800	0.240	0.869	0.658	0.176	0.888	0.132
**ADA(CKSAAP, Binary, AAC)**	**0.778**	**0.801**	**0.222**	**0.912**	**0.720**	**0.132**	**0.925**	**0.150**
SVM(CKSAAP)	0.759	0.801	0.221	0.875	0.668	0.174	0.888	0.130
SVM(Binary)	0.755	0.800	0.245	0.868	0.668	0.169	0.888	0.121
SVM(AAC)	0.627	0.801	0.373	0.744	0.541	0.271	0.820	0.072
SVM(CKSAAP, Binary)	0.858	0.801	0.132	0.935	0.787	0.112	0.942	0.131
SVM(CKSAAP, AAC)	0.656	0.802	0.344	0.780	0.575	0.254	0.848	0.071
SVM(Binary, AAC)	0.664	0.801	0.336	0.775	0.578	0.253	0.850	0.070
**SVM(CKSAAP, Binary, AAC)**	**0.867**	**0.801**	**0.133**	**0.946**	**0.797**	**0.101**	**0.952**	**0.140**
RF(CKSAAP)	0.856	0.801	0.144	0.944	0.789	0.118	0.934	0.148
RF(Binary)	0.843	0.800	0.157	0.941	0.781	0.119	0.930	0.135
RF(AAC)	0.750	0.802	0.250	0.901	0.627	0.165	0.912	0.124
RF(CKSAAP, Binary)	0.877	0.802	0.223	0.928	0.789	0.123	0.954	0.188
RF(CKSAAP, AAC)	0.846	0.801	0.154	0.921	0.778	0.114	0.942	0.147
RF(Binary, AAC)	0.848	0.801	0.152	0.925	0.779	0.120	0.947	0.157
**RF(CKSAAP, Binary, AAC)**	**0.887**	**0.802**	**0.131**	**0.947**	**0.792**	**0.070**	**0.977**	**0.191**

Table S2: Training performance scores at FPR=0.20 for 21 prediction models that were trained with the 1:3 ratio of positive and negative samples.

Predictors	TPR	TNR	FNR	ACC	MCC	MCR	AUC	pAUC
ADA(CKSAAP)	0.765	0.801	0.235	0.878	0.663	0.171	0.892	0.123
ADA(Binary)	0.758	0.800	0.242	0.864	0.659	0.210	0.873	0.119
ADA(AAC)	0.751	0.802	0.259	0.862	0.645	0.197	0.877	0.117
ADA(CKSAAP, Binary)	0.773	0.802	0.227	0.908	0.647	0.141	0.924	0.143
ADA(CKSAAP, AAC)	0.758	0.801	0.242	0.869	0.659	0.188	0.888	0.134
ADA(Binary, AAC)	0.762	0.801	0.238	0.869	0.659	0.183	0.899	0.139
**ADA(CKSAAP, Binary, AAC)**	**0.789**	**0.801**	**0.211**	**0.913**	**0.721**	**0.129**	**0.934**	**0.155**
SVM(CKSAAP)	0.770	0.800	0.230	0.883	0.669	0.171	0.899	0.133
SVM(Binary)	0.766	0.801	0.224	0.879	0.669	0.166	0.899	0.123
SVM(AAC)	0.639	0.801	0.361	0.739	0.544	0.266	0.821	0.079
SVM(CKSAAP, Binary)	0.870	0.802	0.120	0.939	0.789	0.100	0.943	0.133
SVM(CKSAAP, AAC)	0.669	0.801	0.331	0.779	0.577	0.242	0.849	0.072
SVM(Binary, AAC)	0.676	0.801	0.324	0.784	0.579	0.240	0.851	0.073
**SVM(CKSAAP, Binary, AAC)**	**0.877**	**0.802**	**0.123**	**0.948**	**0.799**	**0.091**	**0.954**	**0.144**
RF(CKSAAP)	0.866	0.801	0.114	0.937	0.789	0.106	0.933	0.153
RF(Binary)	0.854	0.800	0.126	0.939	0.784	0.107	0.929	0.146
RF(AAC)	0.764	0.801	0.236	0.907	0.629	0.157	0.915	0.129
RF(CKSAAP, Binary)	0.889	0.801	0.111	0.947	0.789	0.100	0.966	0.198
RF(CKSAAP, AAC)	0.859	0.802	0.141	0.934	0.779	0.112	0.944	0.159
RF(Binary, AAC)	0.858	0.802	0.142	0.936	0.778	0.110	0.949	0.164
**RF(CKSAAP, Binary, AAC)**	**0.899**	**0.801**	**0.121**	**0.959**	**0.795**	**0.060**	**0.978**	**0.199**
