# Supplementary material for: Prediction of serine phosphorylation sites mapping on Schizosaccharomyces Pombe by fusing three encoding schemes with the random forest classifier
Source: Sci Rep. 2022 Feb 16;12:2632. doi: 10.1038/s41598-022-06529-5 (PMC8850546; doi:10.1038/s41598-022-06529-5)
Supplement: Supplementary file 2 — Supplementary Information 2. [file 41598_2022_6529_MOESM2_ESM.pdf]

Supplementary File 2 (File S2)

Two Sample Logo (TSL) for two window sizes 21 and 27:

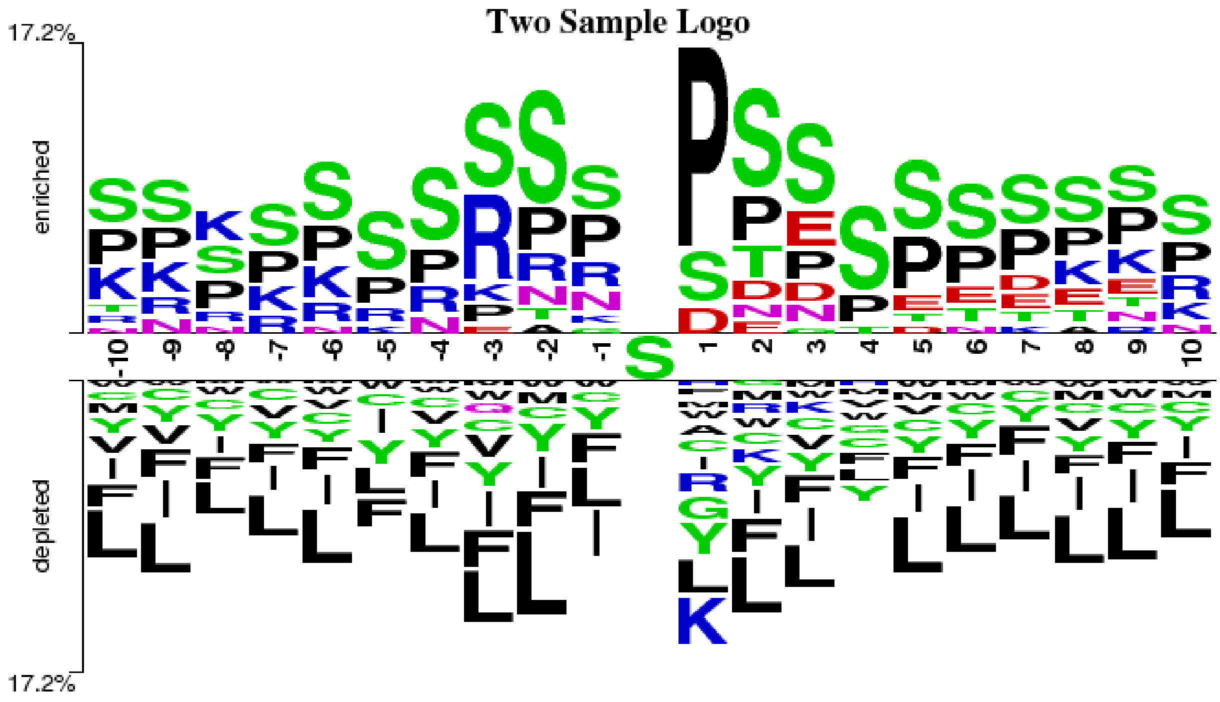

Figure S1: Incidence of amino acid propensities of positive (phosphorylation site) and negative (non-phosphorylation site) windows of size 21 in positions of ~-10 to +10 windows.

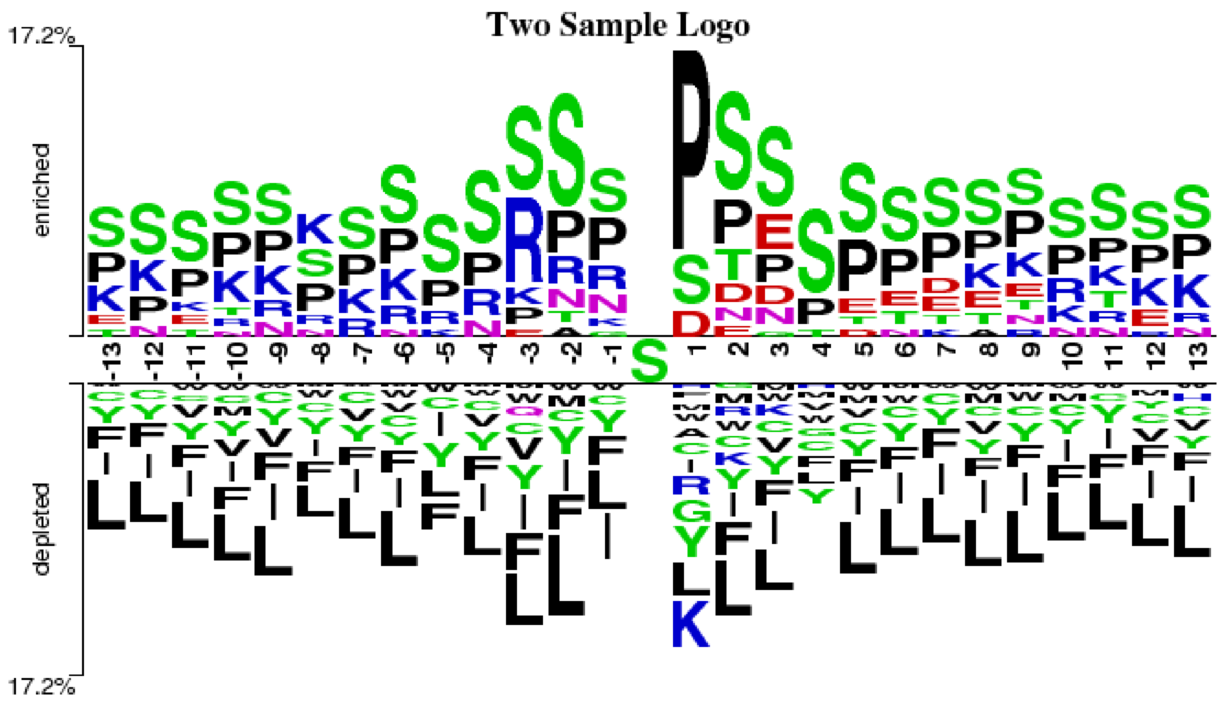

Figure S2: Incidence of amino acid propensities of positive (phosphorylation site) and negative (non-phosphorylation site) windows of size 27 in positions of ~-13 to +13 windows.

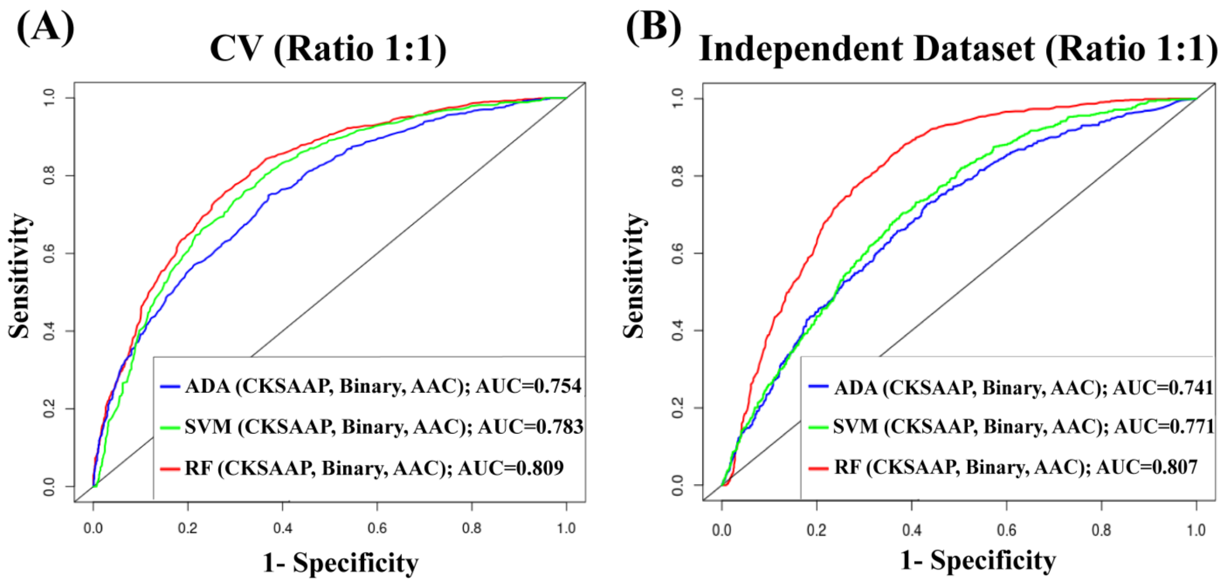

Figure S3: ROC curves for the best prediction models with ADA, SVM, and RF that were trained by the 1:1 ratio of positive and negative samples. (a) Performance curves for the best prediction models with ADA, SVM, and RF by 5-fold CV (b) Independent test performance curves for the best prediction models with ADA, SVM, and RF.

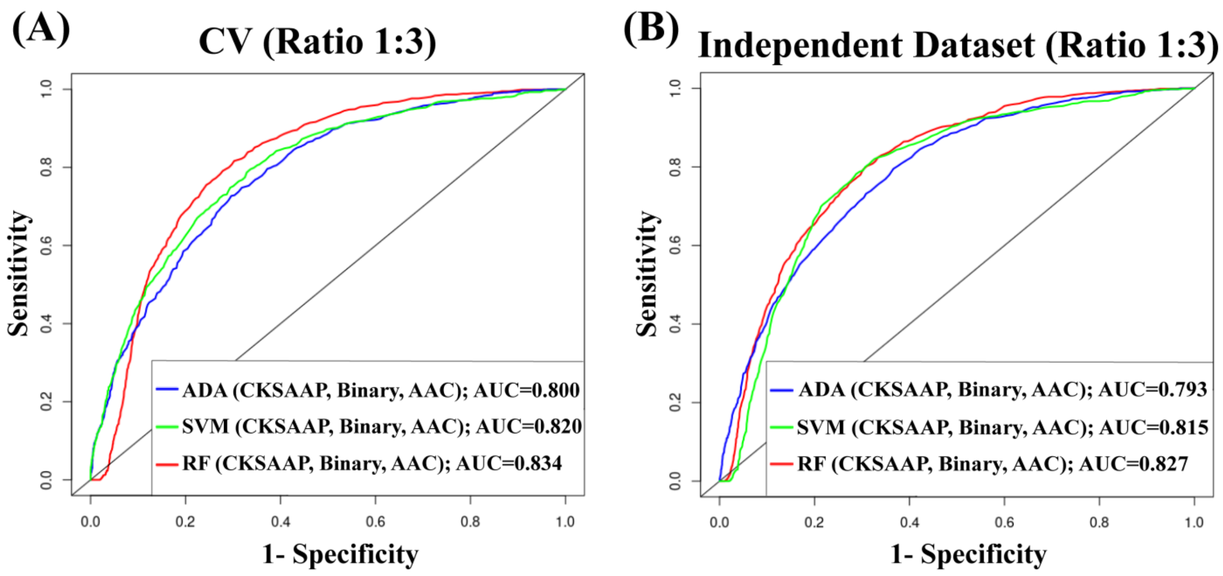

Figure S4: ROC curves for the best prediction models with ADA, SVM, and RF that were trained by the 1:3 ratio of positive and negative samples. (a) Performance curves for the best prediction models with ADA, SVM, and RF by 5-fold CV (b) Independent test performance curves for the best prediction models with ADA, SVM, and RF.
